# Use of technological tools to evaluate handwriting production of the alphabet and pseudocharacters by Brazilian students

**DOI:** 10.6061/clinics/2019/e840

**Published:** 2019-03-19

**Authors:** Giseli Donadon Germano, Simone Aparecida Capellini

**Affiliations:** Departamento de Educacao Especial, Campus Marilia, Universidade Estadual Paulista “Julio de Mesquita Filho” UNESP, Marilia, SP, BR

**Keywords:** Handwriting, Learning, Education, Automation

## Abstract

**OBJECTIVE:**

This study aimed to characterize and compare the handwriting performance of Brazilian students from the 3^rd^ to the 5^th^ grade level of elementary school I with a computerized instrument that allowed the real performance to be observed during the execution of the handwriting.

**METHODS:**

Ninety-five students, aged 8 years to 11 years and 11 months, were assigned the production tasks of handwriting letters and pseudocharacters to assess the variables of latency, letter duration production and movement fluency. The stimulus presentation and the analysis of the movements were analyzed by Ductus software.

**RESULTS:**

In relation to the writing duration, latency and fluency of the alphabet letters, there was a diminution of values from the 3^rd^ to 5^th^ grade. For the comparison between alphabet and pseudocharacter latency, the results indicated a difference between the alphabet letter and its corresponding pseudocharacter, with greater latency for the pseudocharacter. This finding suggests that a motor sequence has not been established, so it cannot be assumed that the production of the alphabet letters was automatic.

**CONCLUSION:**

The results of this study make it possible to verify the interaction failures between the central and peripheral processes, with progression between the 3rd and 5^th^ grade. It also highlights the influence of the lack of systematized teaching of the tracing of letters for Brazilian students since proficiency in calligraphy is critically linked to academic performance. These findings provide a great contribution to Brazilian educational psychology and reflect both educational and clinical practices.

Handwriting; Learning; Education; Automation.

## INTRODUCTION

Handwriting plays a key role in the academic life of students. Despite the development of technologies, handwriting is most often used in everyday activities. Recently, researchers 1-2 have focused on handwriting movements, even when handwriting is not part of the instructional practice for students in the school context and despite the increased use of technology (e.g., tablets and smartphones). One reason is the importance of understanding how movement production not only affects letter forms but also impacts other school achievements, such as spelling and text production, owing to the interaction between these processes 1. Additionally, whatever the underlying cause, handwriting difficulties may lead to reluctance to write, underachievement and low self-esteem 3. Consequently, studies 1-5 found that a certain level of handwriting proficiency enables students to make skillful use of handwriting as a tool to successfully carry out these complex school activities.

Levels of automation have been described as fluent and legible movements, allowing students to release and direct resources to higher-order functions (such as those of a lexical or syntactic nature). Automation is important to letter production, especially because of its impact on future performance in text production 6. The process of acquiring handwriting skills, independent of the orthographic system, implies the use of several motor and cognitive functions 7.

Thus, handwriting production has been described as a hierarchical organization 8, occurring at lower or peripheral levels (e.g., graphomotor planning and execution) but interacting with higher levels (e.g., syntax and semantics). According to the model of Van Galen 8, the central processes refer to orthography, as in writing words, which makes it possible to select and activate orthographic representations 1,9. This activation enables the recovery of the letter components and the necessary organization. An “orthographic” module processes the spelling component of the words, and the motor aspects of letter production are modulated by peripheral processes that regulate the execution of the movement. Subsequently, the “size control” module will process a series of local parameters, such as size, force and speed, allowing adaptations to the local constraints of the writing task so that the letters can be produced efficiently. Once all this information is set up, the “muscular adjustment” module is prepared for movement production. In this way, it is possible to understand a motor program as a sensory-motor map that encodes information about the shape of the letter and the strokes (traces) that are needed for handwriting production 8-10.

In the beginning of the acquisition process, students learn how to write from the production of traces (stroke by stroke). Automation is achieved only when a student acquires enough geometric and cinematic information to produce a letter quickly, ballistically, and with smooth movements. Studies have found that initially, students rely on the coordination of cognitive and perceptual motor processes. Failure in this development can hinder the production of efficient and fluent movements 9-11.

Most studies have concentrated on students from the 1^st^ to 3^rd^ grade level for handwriting analysis 1,9,12-13. This choice is justified because before age 8, or 3^rd^ grade level, letter production is relatively slow 12, as the interaction between spelling and motor processes can still be limited, and most graphomotor gestures require extreme control and close sensory guidance 8-10. These motor programs will be stored in long-term memory, and as the students practice these movements, they become automatized. As this process occurs, there is a diminution of sensory feedback and an increase in movement speed. This development requires a long process that might end at approximately 10-11 years old 14.

Studies that evaluate this relationship between letter production and its automation are rare in the Brazilian context. Therefore, it is important to evaluate measures of these aspects because they enable the observation of underlying specific events that characterize the movement. The use of tablets and software for the evaluation of handwriting enables the evaluation of such events during the execution of writing production through an analysis of the spatial trajectory 15. These technologies supply an unbiased and direct way of evaluating dynamic handwriting subprocesses (e.g., writing time duration, airtime, or fluency) 16. However, there are no Brazilian studies that use computerized evaluations through digital surfaces (tablets) to evaluate manual handwriting.

Therefore, this study presents the hypothesis that the lack of handwriting practices in the school context might influence the process of handwriting automation. Students who have difficulty in the automatic handwriting production of letters may be at risk of delays in academic progress during their early school years 17. Thus, this study is justified due to the lack of specific assessment tools based on computerized analytical measures and the need to demonstrate whether this interaction between the central and peripheral processes of letter handwriting occurs in the Brazilian population.

The study of variables, such as reaction time and movement production time, using graphic tablets enables the analysis of motor programming 18 and the nature of the motor programs involved in writing or the size of the basic unit (letters or the constitutive characteristics of letters) 15. Therefore, due to the impact of legibility and fluency of handwriting, which are related to automation, on students' academic and social activities, this study aimed to characterize and compare the performance of Brazilian students from the 3rd to the 5^th^ grade level of elementary school I by using a technological tool to assess handwriting tasks related to letters and pseudocharacters based on the variables of latency, letter duration production and movement fluency.

## METHODS

This study was approved by the Research Ethics Committee of the Faculty of Philosophy and Sciences, São Paulo State University “Júlio de Mesquita Filho” (FFC/UNESP), Protocol No. 1117/2014.

A total of 95 students, aged from 8 years to 11 years and 11 months and of both sexes, were divided into Group I (GI, composed of 27 students from the 3^rd^ grade level of elementary school); Group II (GII, composed of 37 students from the 4^th^ grade level of elementary school) and Group III (GIII, composed of 31 students from the 5^th^ grade level of elementary school). The parents and/or guardians of all the participants signed an informed consent form. Exclusion criteria for participation in the study were as follows: students with sensory, motor, or cognitive impairment; students whose parents and/or guardians did not sign the informed consent form; and students with unsatisfactory academic performance, assessed as grades below five in Portuguese and math tests conducted by the teachers in the classroom. These criteria are based on the teachers' reports in the SARESP Pedagogical Reports 19. The inclusion criteria were as follows: students whose parents and/or guardians signed the informed consent form; students without sensory, motor, or cognitive impairment, according to information from the school records; and students with satisfactory academic performance, assessed as grades above five in Portuguese and math tests. These criteria are based on the teachers' reports in the SARESP Pedagogical Reports 19. All participants were right-handed and were native Brazilian Portuguese speakers. Additionally, as referred to in international studies 1,12,14, by the age of 8, handwriting production is relatively slow, and the development of automation might end at approximately age 10-11.

To perform the procedures described below, a notebook with installed software (adapted version) was used, attached to a digitalized table (Wacom Intuos 5). The stimulus presentation and the movements were analyzed by Ductus software 20. The students were assigned two tasks: the first consisted of writing capital letters, and the second consisted of copying pseudocharacters. The students were asked to write the letters of the alphabet in uppercase format 6,16.

For this study, three measures were selected. First, latency (measured in milliseconds) referred to the difference between the time that a stimulus was presented and the moment that a student started writing (with pen pressure >0). This measure favors the comprehension of the time that the students have to prepare movements before beginning to write a letter. Second, letter duration production, measured in milliseconds (ms), referred to the time needed by the students to write each letter of the alphabet. Third, the movement fluency measure (measured in cm/ms) referred to the number of absolute velocity peaks of the movements produced in writing each letter 1,9-10. To compare the measures of duration and fluency, we divided the total movement time and the number of velocity peaks by the number of strokes in each item. The number of strokes for a given item was the sum of the strokes in each letter, and a normalization procedure was performed by the authors 21-22.

## RESULTS

The results were statistically analyzed using the SPSS V20, Minitab 16 and Excel Office 2010 software. The *p*-values were considered statistically significant at the adopted significance level (0.05), indicated by asterisks (*). Tables 1 to 3 show the distribution of the letters of the alphabet for the variables latency, duration and fluency of letter production in the comparison between the groups GI, GII and GIII and multiple comparisons.

In Table 1, a significant difference was observed for latency in the comparison between the groups for the letters E, H, I, R and S, which was verified in relation to the means by higher scores for GI, followed by GII and GIII (GI> GII> GIII). In the multiple comparisons, the letters E and S showed a significant difference between the *p*-values in the comparisons between GI and GII and GI and GIII, with a higher mean latency for GI. These findings indicate a decrease in latency as the students reach later school years, suggesting that GI students experience greater difficulty in tracing, and it can be inferred that beginning in the 5^th^ grade, these processes become more automated, and access therefore becomes faster. For the letters H, I and R, it was possible to verify that there was a significant difference between the *p*-values in the comparison between the GI and GIII for the same variable; the decrease in latency between the groups may indicate a possible automation of the execution of the letter (handwriting) in the 5^th^ grade (GIII).

In Table 2, for the duration of the letter production, a significant difference was observed in the comparison between groups (mean GI>GII>GIII) for most letters (C, D, E, F, G, H, J, K, M, N, O, P, Q, R, S, T, V, W, and X), with the exception of the letter B (mean GII> GI> GIII). These findings suggest that for most letters, there is a progression of the advancement of education and a reduction in time, evidencing an automation of the motor planning for these letters. Regarding the multiple comparisons, for the letters C, E, F, G, K, Q, R, T, and V, a significant difference was observed between the *p*-values for GI and GIII and GII and GIII, suggesting that there was a reduction in the time needed for the execution of these letters as the students reached later school years, evidencing automation or greater interaction between the central and peripheral processes. For the letters D, H, M, N, O, P, W and X, it was possible to verify that there was a significant difference between the *p*-values for GI and GIII for the same variable; the decrease in duration between the groups may indicate a possible automation of the execution of the letters between the 4^th^ (GII) and 5^th^ (GIII) grades. For the letter J, there was a significant difference between the *p*-values in the comparison between GI and GII and GI and GIII, although no differences were observed between GII and GIII. These findings indicate that there was no decrease in duration as the students reached later school years, suggesting that GI students experience greater difficulty in the interaction between the central and peripheral processes. It can be inferred that beginning in the 4^th^ and 5^th^ grades, these processes become more automated and thus can be accessed faster.

In Table 3, regarding the relation to fluency of letter production a significant difference was verified in the comparison of the groups. For most of the letters (B, C, D, E, F, G, J, K, M, O, P, Q, R, S, T, W and X), in relation to the means, higher scores were found for GI, followed by GII and GIII (GI> GII> GIII); that is, the GI students (3^rd^ grade) presented the highest number of speed peaks compared to GIII (5^th^ grade). Although GI students are more exposed to reading and writing practices with materials that primarily use capital letters, which favors the formation of visual memory, this memory is accessed more quickly in later school years, which influences handwriting production and makes students more agile in the execution of these letters. Regarding multiple comparison, for the letters D, E, G, K, O, R, S, T, W and X, there was a significant difference between the *p*-values in the comparison between the GI and GIII groups for the same variable, indicating that there was a decrease in the number of absolute speed peaks (fluency) between the groups.

Students from GI were faster than those from GIII; however, GI presented more disfluency peaks, that is, less inaccuracy of movements, meaning that they decrease the speed of handwriting but possibly present better handwriting quality. For the letters C, F, M and Q, a significant difference was observed between the *p*-values in the comparison between GI and GIII and GII and GIII, suggesting that there is a decrease in the fluency of letter execution as the students reach later schooling years. For the letters J and D, a significant difference was verified in the comparison between GI and GII and GI and GIII for the same variable, indicating a decrease in fluency between the groups; in addition, GI students presented faster movements than GIII students. As observed in the previous tables, these processes become more automated for GIII students (based on the latency and duration parameters). However, some slowness concerning fluency is observed from GI to GIII, suggesting a possible disfluency of the movement, or overload, due to the task or interference in the interaction between the central and peripheral processes. Nonetheless, as students reach later school years, there is a greater concern with other issues, such as text production, and less emphasis on isolated letter-writing practices. In relation to the fluency variable of letter production, students may be influenced by educational practices.

Additionally, a comparison was performed between the three groups for the pseudocharacter stimuli in relation to the latency time. There was no significant difference between the groups, suggesting that students from GI, GII and GIII may not have the so-called “motor maps” for the letters of the alphabet. This suggestion could be inferred, as the pseudocharacters represent the same number of strokes as the letters of the alphabet; that is, the realization of similar movements for letters that can be retrieved from the long-lasting memory.

**Figure 1:**
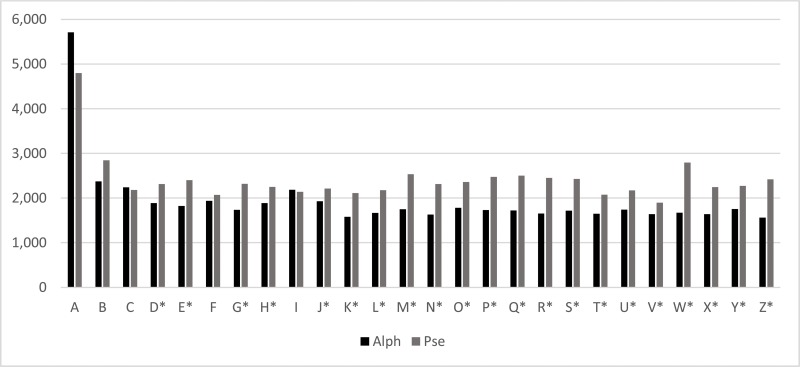
Comparison between the alphabet (Alph) and pseudocharacters (Pse) for latency. Student's paired t-test (*p<0.05).

Finally, a comparison between the letters of the alphabet and pseudocharacters was performed for the latency variable for each letter and its corresponding pseudocharacter using Student's paired t-test. This comparison was performed because nonlinguistic stimuli tasks might involve control skills and recovery of memory movements (Figure 1).

In Figure 1, it is possible to observe a significant mean difference between the latencies of the alphabet and the corresponding pseudocharacters in most of the letters except for A, B, C, F and I. For letters that presented a significant difference, it was observed that the mean latency was higher for pseudocharacters than than for the alphabet letters. The pseudocharacter stimuli have the same number of strokes as the letters of the alphabet, with changes in their arrangement (changed angles between strokes, inversion, etc.). These findings suggest that the students in this study had not developed a motor program in their long-term memory. A difference between an alphabet letter and its corresponding pseudocharacters suggests that a motor sequence has not been established; thus, it cannot be assumed that the production of the alphabet letters was automatic. Automation would be related to the implicit motor learning of sensory-motor associations, which is progressively more complex.

## DISCUSSION

The results of this study indicated that the students presented difficulties in the production of the letters of the alphabet in the initial school years but progressed to automation by the 5^th^ grade in relation to the parameters of latency, duration and fluency. These results suggest that students begin to access the motor programs to write the alphabet, decrease the time interval after the presentation of the stimulus (latency), reduce the handwriting production time (duration) and improve the handwriting speed (fluency).

In relation to the handwriting duration of the alphabet letters, the GI students of this study needed more time. However, it is possible to observe a reduction in the duration of the production of letters from the 3^rd^ (8 years old) to 5^th^ (11 years old) grade level. Such findings are in accordance with international studies 13,15, that studied the handwriting production of students from ages 6 to 11 (1^st^ to 5^th^ grade) and demonstrated that at the early stages of writing acquisition, students write letters stroke by stroke, leaving little room for the planning and revision processes 15.

Through practice, they begin to join the traces, and handwriting becomes faster and smoother. From the moment when the students decide which letter to use, a motor output program begins, which is influenced by the allograph variation. Each allograph represents different motor programs related to font and size. Other authors 22, have referred to this process of the production of letters, emphasizing that during writing acquisition, students learn a set of rules for letter production. These rules can be understood as an “action grammar,” which has specific characteristics, such as the location and direction in which to produce writing and when the movements should start and end. Initially, in the early years of literacy, these rules are applied and accessed during the production of letters and carry a strong cognitive load. Then, the automation of writing occurs and reduces the cognitive load. Thus, it can be inferred that the automation of letter production occurs when cognitive resources are allocated to other processes that compose writing, such as spelling and elaboration of the production of sentences and text, which occur in 5^th^ grade level practice.

In relation to fluency, for the letter production of the alphabet, the students in this study presented lower values in the 5^th^ grade level of elementary school I, with a small improvement in the 4^th^ grade and regression in the 5^th^ grade. Thus, there was a decrease in the speed of handwriting production, but the students may have become more precise in the execution of their movements. To have a proper movement flow, the students must recover and maintain the forms of production from the motor program stored in long-term memory.

Studies 22,24 have shown that when students become more fluent, that is, when their movements become more precise, there is a cognitive transition in which the working memory is no longer used for the production of specific forms of letters and therefore is released for text production. An additional aspect of handwriting fluency is the physical act of writing letters. With the improvement and learning of physical gestures to write letters, the speed and facility of writing increase. However, this process was not the case for the students in this study. The results of this study suggest that to program a movement properly, the students decreased their production speed to achieve better movement accuracy. Therefore, the findings of this study corroborate others 25-26 that indicating that the influence of increased cognitive demands on movement production can interrupt motor planning and the execution of the manual actions, such as handwriting, of young students.

According to international studies, in grades 4-6 (9 to 12 years old), transcription becomes more automated, enabling advanced macrostructural planning (i.e., planning of the main items of the superstructure and grouping of the microstructural elements) to emerge and post-translation revision to take place at the text level 24. Additionally, graphonomic research in adults has identified several factors affecting handwriting fluency that are related to increased attention when writing a word or when an attempt to write neatly has a detrimental effect on automation 6.

One aspect to be considered in the Brazilian context is the absence of systematic teaching of the movements of writing letters and the changes that occurred in the mid-1980s, when the teaching of the letter-writing movements was relegated to a secondary plane and the aspects of language were emphasized instead. Thus, the impairment of fluency in the 3^rd^ grade may be influenced not only by the acquisition processes and memory demand for movements but also by the lack of adequate instruction. This finding is important because performance in word and text production can be impacted.

As mentioned by Thibon et al. 22, to learn how to write a letter, it is necessary to memorize a motor sequence in a specific order. Students in this study began to automate the production of letters at approximately the age of 10. Initially, their movements were related to the production of strokes with greater cognitive overload, and there was a progressive decrease in overload and distress by the end of the 5^th^ grade.

Consequently, there is a motor program for each letter that contains information about it, such as shape, size, and direction and order of the strokes. This program should be activated in the long-term memory each time a student needs to write a letter. In this way, the number of strokes of a given letter should no longer be responsible for the cognitive load of movement execution. The automation of writing production is achieved when there is no more cognitive overload for letter production, and handwriting then becomes an instrument of communication 22.

As a result, studies have emphasized the importance of the role of teachers in instruction in letter movements, as the development of programs for the production of letters takes place when teachers explicitly explain the production procedures. Teachers contribute to the construction of the “grammar of action” that provides information such as the location at which to start writing and the sequence students need to follow to write a letter 22,24,27.

However, it is important to highlight the lack of systematized teaching for the transition from writing in capital letters to writing in cursive. The National Pact for Literacy in the Right Age 28-29 indicates that students should have more experience with written language and not worry about the development of motor strategies; thus, the teaching of cursive begins in the 3^rd^ grade of elementary school I and is required until the end of the 5^th^ grade. In this way, the students in this study may have presented higher fluency in the execution of letters of the alphabet in the 3^rd^ grade because of greater exposure (reading books) and because capital letters are preferred by Brazilian teachers.

Regarding the comparison of the latency time between the production of writing alphabet letters and the corresponding pseudocharacters, the students in this study needed more time for the execution of the second stimulus. Such latency results indicate the activation of central and peripheral processes before beginning to write, but the interaction is incomplete because there was a difference between the alphabet letter and the corresponding pseudocharacter, with greater latency for the pseudocharacter. This finding suggests that a motor sequence has not been established, so it cannot be assumed that the production of the alphabet letters was automatic.

Studies 30-31 have reported that early literacy students tend to use semiographic strategies; that is, they start school activities with discontinuous graphic features, a set of pseudocharacters, some known letters (of their own first name or familiar words) and numbers scattered on a sheet. Initially, most students produce continuous and discontinuous pictorial traces as well as pseudocharacters, but this trend decreases over time. The students then use the semiographic strategy, that is, traces as pseudocharacters, which can also be called ideograms. Therefore, the students begin to assign a symbolic graphic meaning to the characters. However, studies indicate that the process memory (movement sequence to be coded) is present for the execution of the pseudocharacter copying task because the characters have the same number of letter strokes 22,30-31.

Thus, the results of this study indicate that, for Brazilian students, there has not yet been a complete automation of alphabet letter production, suggesting that the central and peripheral processes do not occur simultaneously. These findings suggest that these students may have difficulty in producing texts in subsequent school years. A lack of practice was observed among the Brazilian students and may indicate interaction failures between the central and peripheral process variables, as verified for the latency variables. In regard to Brazilian students, the continuity of studies that analyze the production of words is necessary to verify the impact of these processes on lexicon recovery and writing production, as there is no formal systematized calligraphic teaching.

## CONCLUSION

The results of this study make it possible to verify the interaction failures between the central and peripheral processes, with progression between the 3^rd^ and 5^th^ grades. The study also highlights the influence of the lack of systematized teaching on the tracing of letters for Brazilian students since proficiency in calligraphy is critically linked to academic performance. This finding emphasizes the importance of identifying and supporting those with handwriting difficulties to ensure that they are able to achieve their potential. As a limitation of this study, we can indicate the difficulty in evaluating students from the 1st to 3^rd^ year of school who still have not achieved the full domain of letter traces, as foreseen by the national curriculum. These findings are in line with our hypothesis. The results of this study also suggest the necessity of a discussion of the implementation of educational programs that favor motor maps. It should be noted that for Brazilian students, there is a need for the continuity of studies that analyze the production of words to verify the impact of these processes in the recovery of the lexicon and the production of writing, as there is no formal systematized calligraphic teaching.

## AUTHOR CONTRIBUTIONS

Germano GD designed the experiment and project, collected and interpreted the data and wrote the main manuscript. Capellini SA provided technical support and conceptual advice, wrote the main manuscript and approved the final version of the manuscript to be published. All authors discussed the results and implications and commented on the manuscript at all stages.

## Figures and Tables

**Table 1:** Comparisons of latency performance.

		Mean (±SD)	*p*-value	Multiple comparisons	*p*-value
Letter E	GI	2.193 (893)	0.003*	GI≠GII	0.018*
	GII	1.729 (544)		GI≠GIII	0.004*
	GIII	1.614 (539)		GII≠GIII	0.756
Letter H	GI	2.351 (1.495)	0.005*	GI≠GII	0.205
	GII	1.906 (939)		GI≠GIII	0.004*
	GIII	1.455 (479)		GII≠GIII	0.173
Letter I	GI	2.507 (1.157)	0.028*	GI≠GII	0.875
	GII	2.356 (1.568)		GI≠GIII	0.037*
	GIII	1.709 (631)		GII≠GIII	0.078
Letter R	GI	1.938 (802)	0.019*	GI≠GII	0.225
	GII	1.645 (760)		GI≠GIII	0.014*
	GIII	1.410 (485)		GII≠GIII	0.352
Letter S	GI	2.076 (881)	0.003*	GI≠GII	0.040*
	GII	1.658 (605)		GI≠GIII	0.003*
	GIII	1.474 (500)		GII≠GIII	0.496

Test ANOVA (*p<0.05).

Test ANOVA, Tukey multiple comparisons (post hoc) (*p<0.05).

**Table 2:** Comparisons of duration performance.

		Mean (SD)		Multiple comparisons	*p*-value		Mean (SD)	*p*-value	Multiple comparisons	*p*-value
Letter B	GI	359.5 (159)	0.011*	GI≠GII	0.875	Letter N	367.5 (182.1)	0.034*	GI≠GII	0.595
	GII	380 (176)		GI≠GIII	0.07		329.4 (156.4)		GI≠GIII	0.03*
	GIII	262.4 (154.6)		GII≠GIII	0.012*		262.3 (123.2)		GII≠GIII	0.181
Letter C	GI	559.7 (265.9)	0.009*	GI≠GII	0.991	Letter O	662 (369.2)	0.014*	GI≠GII	0.59
	GII	551.6 (273.7)		GI≠GIII	0.024*		589.2 (264.3)		GI≠GIII	0.013*
	GIII	385.1 (192.6)		GII≠GIII	0.019*		439.6 (247.6)		GII≠GIII	0.096
Letter D	GI	432.4 (193)	0.024*	GI≠GII	0.316	Letter P	502.2 (237.5)	0.002*	GI≠GII	0.062
	GII	368.3 (161.7)		GI≠GIII	0.018*		390.2 (192)		GI≠GIII	0.001*
	GIII	304.9 (169.7)		GII≠GIII	0.296		318.3 (146.5)		GII≠GIII	0.282
Letter E	GI	487.4 (180.5)	0.001*	GI≠GII	0.561	Letter Q	505.6 (259.5)	0.003*	GI≠GII	0.943
	GII	440.8 (200.3)		GI≠GIII	0.001*		525 (260.3)		GI≠GIII	0.02*
	GIII	310.4 (148.7)		GII≠GIII	0.01*		336.3 (168.7)		GII≠GIII	0.004*
Letter F	GI	492.5 (195.3)	<0.0018*	GI≠GII	0.954	Letter R	437.5 (228.5)	0.003*	GI≠GII	0.598
	GII	478.6 (221.7)		GI≠GIII	0.001*		390.5 (215.9)		GI≠GIII	0.003*
	GIII	308.1 (125.6)		GII≠GIII	0.001*		266.2 (107.5)		GII≠GIII	0.025*
Letter G	GI	563 (246.4)	0.010*	GI≠GII	0.734	Letter S	371.4 (127.8)	0.046*	GI≠GII	0.873
	GII	521.4 (238)		GI≠GIII	0.012*		352.1 (193.6)		GI≠GIII	0.056
	GIII	394.2 (163.4)		GII≠GIII	0.05*		277.4 (111.7)		GII≠GIII	0.116
Letter H	GI	530.4 (203)	0.010*	GI≠GII	0.455	Letter T	602.7 (281.2)	0.003*	GI≠GII	0.664
	GII	468.2 (225.4)		GI≠GIII	0.008*		551.8 (225.5)		GI≠GIII	0.003*
	GIII	366.3 (176.2)		GII≠GIII	0.106		397 (190.8)		GII≠GIII	0.020*
Letter J	GI	708.2 (419.9)	0.001*	GI≠GII	0.019*	Letter V	590.8 (269.7)	0.020*	GI≠GII	0.997
	GII	510.3 (203.5)		GI≠GIII	0.001*		585 (409.1)		GI≠GIII	0.045*
	GIII	433.7 (205.5)		GII≠GIII	0.509		391.7 (187.7)		GII≠GIII	0.034*
Letter K	GI	550.2 (237.7)	0.001*	GI≠GII	0.385	Letter W	443.2 (208.9)	0.017*	GI≠GII	0.854
	GII	477.5 (253.9)		GI≠GIII	0.001*		419.3 (172.5)		GI≠GIII	0.024*
	GIII	336.2 (132.8)		GII≠GIII	0.024*		319 (148)		GII≠GIII	0.056
Letter M	GI	441.4 (173.7)	0.016*	GI≠GII	0.871	Letter X	610.6 (314.2)	0.001*	GI≠GII	0.173
	GII	420 (176.4)		GI≠GIII	0.023*		497.1 (246.4)		GI≠GIII	0.001*
	GIII	322.4 (154.2)		GII≠GIII	0.051		363.2 (175.1)		GII≠GIII	0.074

Test ANOVA (*p<0.05).

Test ANOVA, Tukey multiple comparisons (post hoc) (*p<0.05).

**Table 3:** Comparisons of fluency performance.

		Mean (SD)	*p*-value	Multiple comparisons	*p*-value		Mean (SD)	*p*-value	Multiple comparisons	*p*-value
Letter B	GI	2.46 (1.1)	0.030*	GI≠GII	0.897	Letter O	5.22 (3.37)	0.031*	GI≠GII	0.538
	GII	2.61 (1.52)		GI≠GIII	0.127		4.49 (2.6)		GI≠GIII	0.026*
	GIII	1.79 (1.16)		GII≠GIII	0.03*		3.32 (2.22)		GII≠GIII	0.192
Letter C	GI	4.24 (1.88)	0.013*	GI≠GII	0.834	Letter P	3.49 (1.68)	0.002*	GI≠GII	0.030*
	GII	3.95 (2.29)		GI≠GIII	0.018*		2.55 (1.43)		GI≠GIII	0.002*
	GIII	2.76 (1.8)		GII≠GIII	0.047*		2.15 (1.21)		GII≠GIII	0.493
Letter D	GI	3.21 (1.63)	0.010*	GI≠GII	0.204	Letter Q	3.6 (2.41)	0.012*	GI≠GII	0.948
	GII	2.58 (1.36)		GI≠GIII	0.007*		3.45 (2.12)		GI≠GIII	0.023*
	GIII	2.02 (1.4)		GII≠GIII	0.262		2.22 (1.15)		GII≠GIII	0.030*
Letter E	GI	2.7 (1.37)	0.024*	GI≠GII	0.455	Letter R	2.89 (1.51)	0.005*	GI≠GII	0.522
	GII	2.32 (1.32)		GI≠GIII	0.019*		2.53 (1.44)		GI≠GIII	0.005*
	GIII	1.8 (1.03)		GII≠GIII	0.199		1.79 (0.8)		GII≠GIII	0.051
Letter F	GI	2.98 (1.43)	0.002*	GI≠GII	0.844	Letter S	2.85 (1.21)	0.019*	GI≠GII	0.536
	GII	2.78 (1.64)		GI≠GIII	0.004*		2.51 (1.49)		GI≠GIII	0.016*
	GIII	1.78 (0.83)		GII≠GIII	0.009*		1.92 (0.94)		GII≠GIII	0.135
Letter G	GI	4.04 (1.94)	0.019*	GI≠GII	0.722	Letter T	3.5 (2.04)	0.032*	GI≠GII	0.606
	GII	3.7 (1.71)		GI≠GIII	0.021*		3.11 (1.57)		GI≠GIII	0.028*
	GIII	2.81 (1.5)		GII≠GIII	0.086		2.39 (1.21)		GII≠GIII	0.166
Letter J	GI	4.93 (2.97)	0.002*	GI≠GII	0.015*	Letter W	2.51 (1.32)	0.029*	GI≠GII	0.592
	GII	3.43 (1.57)		GI≠GIII	0.003*		2.24 (1.13)		GI≠GIII	0.026*
	GIII	3.06 (1.6)		GII≠GIII	0.747		1.74 (0.83)		GII≠GIII	0.161
Letter K	GI	2.98 (1.78)	0.015*	GI≠GII	0.619	Letter X	3.78 (2.42)	0.002*	GI≠GII	0.1
	GII	2.6 (1.83)		GI≠GIII	0.014*		2.82 (1.83)		GI≠GIII	0.002*
	GIII	1.8 (0.88)		GII≠GIII	0.092		2.06 (1.01)		GII≠GIII	0.203
Letter M	GI	2.85 (1.38)	0.002*	GI≠GII	0.381					
	GII	2.45 (1.24)		GI≠GIII	0.002*					
	GIII	1.74 (0.97)		GII≠GIII	0.048*					

Test ANOVA (*p<0.05).

Test ANOVA, Tukey multiple comparisons (post hoc) (*p<0.05).
